# VEGF promotes migration and invasion by regulating EMT and MMPs in nasopharyngeal carcinoma

**DOI:** 10.7150/jca.46429

**Published:** 2020-10-21

**Authors:** Li Chen, Guoxiang Lin, Kaihua Chen, Renba Liang, Fangzhu Wan, Chuxiao Zhang, Ge Tian, Xiaodong Zhu

**Affiliations:** 1 Department of Oncology, Affiliated Wuming Hospital of Guangxi Medical University, Nanning, Guangxi, 530010, People's Republic of China.; 2 Department of Radiation Oncology, Guangxi Medical University Cancer Hospital, Nanning, Guangxi 530021, People's Republic of China.; 3 Guangxi Key Laboratory of Early Prevention and Treatment for Regional High Frequency Tumor, Guangxi Medical University, Nanning, Guangxi 530021, People's Republic of China.

**Keywords:** nasopharyngeal carcinoma, VEGF, invasion, migration

## Abstract

**Background:** Vascular endothelial growth factor (VEGF) is an important pro-angiogenic factor. Accumulating data have indicated that VEGF is involved in tumour metastasis. However, the mechanism through which VEGF regulates nasopharyngeal carcinoma (NPC) metastasis is largely unknown. This study aimed to examine the biological function of VEGF in NPC metastasis and its underlying mechanism.

**Methods:** We used western blotting and qPCR to examine the difference in VEGF expression between NPC cells and the immortalized nasopharyngeal epithelial cell line NP69. Wound healing assays, transwell assays and animal experiments were used to further verify the role of VEGF in the invasion and migration of NPC cells. The protein levels of the epithelial-mesenchymal transition (EMT) and matrix metalloproteinase (MMP) family were analysed by immunofluorescence (IF) and western blotting. Enzyme-linked immunosorbent assay (ELISA) and transwell assays were used to determine whether VEGF enhanced the invasion and migration of NPC cells in an autocrine manner. Western blotting was used to examine how autocrine VEGF-VEGFR2 signalling regulated EMT and MMPs.

**Results:** We observed higher levels of VEGF in NPC cells than that in NP69 cells and identified an association between high VEGF levels and tumour invasion and migration. Mechanistically, the VEGF-mediated increase in EMT markers, MMP2 and MMP9 promoted NPC cell invasion and migration**.** Additionally, NPC cells secreted VEGF to promote cell invasion, migration and angiogenesis. Autocrine VEGF-VEGFR2 signalling increased ERK1/2 phosphorylation, promoted EMT process and MMPs at the indicated times.

**Conclusion:** This study revealed that VEGF plays a role in controlling NPC cell metastasis by regulating EMT markers and MMPs in an autocrine manner.

## Introduction

Nasopharyngeal carcinoma (NPC) is a common cancer of the head and neck in Southeast Asia and is mainly treated by radiotherapy 1. Most newly diagnosed NPC patients (75-90%) have locally advanced disease that is usually accompanied by cervical lymph node metastasis 2. Currently, metastasis causes a large proportion of treatment failure in NPC 3. Therefore, clarifying the molecular mechanisms of NPC cell metastasis and finding effective targeted therapies are important ways to increase the survival rate.

Because tumour cells rapidly proliferate, there are high levels of blood vessel growth to provide oxygen and nutrition for rapidly growing tumour cells 4. Normal angiogenesis involves a dynamic balance between angiogenic factors and antiangiogenic factors; once the balance is disrupted, it can easily lead to tumour growth and metastasis 4, 5. Vascular endothelial growth factor (VEGF) is a cytokine that promotes angiogenesis *in vivo* 6, and many studies have shown that VEGF contributes to tumorigenesis and metastasis 7-9. A clinical trial suggested that VEGF is related to poor prognosis and tumour-node-metastasis (TNM) staging in NPC 10. Nevertheless, the specific mechanism by which VEGF affects NPC metastasis is poorly understood.

In the last decade, many studies have demonstrated that VEGF secreted by cancer cells stimulates cell proliferation and metastasis not only in a paracrine manner but also via the autocrine activation of VEGF receptors (VEGFR1/2/3) 11-18. The binding of VEGF to VEGFR2 is the key step in tumour angiogenesis 12, 19, and autocrine VEGF-VEGFR2 signalling promotes tumour progression and migration^16^, ^20^, ^21^. The matrix metalloproteinase (MMP) family plays an important role in the process of tumour metastasis. Among these proteins, MMP2 and MMP9 degrade collagen and remove matrix molecules from the extracellular matrix and basement membrane 22. Furthermore, VEGF binds to the receptor that activates VEGFR2 and promotes the secretion of MMPs, which leads to tumour metastasis 22, 23. Epithelial-mesenchymal transition (EMT) is a process in which epithelial cells lose their epithelial properties and acquire mesenchymal properties 24. During EMT, the basement membrane and polarization of tumour cells are destroyed, resulting in increased cell motility 25. In prostate cancer, VEGF induces EMT process through an autocrine loop, which leads to cancer cell metastasis 26. Therefore, it will be interesting to examine whether VEGF regulates EMT markers and MMPs in an autocrine manner to promote NPC cell migration and invasion.

To answer this question, we silenced VEGF expression in NPC cell lines and detected the effects on VEGF secretion and metastasis. We demonstrated that the knockdown of VEGF inhibited the migration and invasion of NPC cells *in vitro* and *in vivo* by suppressing the expression of EMT markers and MMPs. In addition, we also found that the mechanism of action of VEGF is related to an autocrine pathway that involves VEGFR2 binding. Therefore, in the current study, we found that the autocrine VEGF-VEGFR2 signalling pathway promotes NPC metastasis by enhancing EMT and MMP expression. These data provide novel viewpoints for the study of metastatic mechanisms in NPC.

## Materials and methods

### Cell lines and transfection

NPC cell lines (5-8F and CNE-2) and the immortalized nasopharyngeal epithelial cell line NP69 were maintained by our laboratory. CNE-2 and 5-8F cells were cultured in DMEM supplemented with 10% foetal bovine serum (FBS). NP69 cells were cultured in keratinocyte serum-free medium (KSFM) supplemented with rEGF. These cells were cultured in a 37°C incubator with 5% CO_2_. GV248-Puromycin-EGFP-shRNA-VEGF lentiviral vectors (Genechem, Shanghai, China) were transfected into NPC cells.

### qPCR analysis

RNAiso Plus reagent (Takara, Dalian, Japan) was used to isolate total RNA from NPC cells and NP69 cells, and total RNA was reverse transcribed into cDNA. A 20 μl qPCR system was prepared by combining cDNA with primers and then placing the mixture in a PCR instrument for qPCR. The relative quantitative analysis of mRNA levels was conducted by the 2^-ΔΔct^ method. The following primer sequences were used: (5′→3′): VEGF-F: TCACAGGTACAGGGATGAGGACAC, VEGF-R: CAAAGCACAGCAATGTCCTGAAG; GAPDH-F: CAGGAGGCATTGCTGATGAT, and GAPDH-R: GAAGGCTGGGGCTCATTT.

### Western blot analysis and immunofluorescence (IF)

RIPA cell lysis buffer (Beyotime, Beijing, China) was used to isolate total protein from cells. The quantitative detection of proteins was conducted by a BCA (Beyotime, Beijing, China) method. After electrophoresis, the protein samples were transferred to PVDF membranes, sealed, and then incubated with primary and secondary antibodies. The protein bands were visualized with enhanced chemiluminescence (ECL) and analysed with ImageJ software. For IF, cells were inoculated on coverslips, fixed with 4% paraformaldehyde, and blocked with Immunol staining blocking buffer. Next, the cells were incubated with primary and secondary antibodies. Finally, nuclei were counterstained with a DAPI solution. Cell staining was observed by an EVOS FL Auto microscope (Life Technologies, USA).

### Wound healing assay

NPC cells were resuspended in serum-free medium, added to a culture insert (1×10^3^ cells/insert), and wounds were made using the cell culture inserts (Ibidi, Germany). The cells were cultured in a 37°C incubator with 5% CO_2_ for 36 h. Wound healing was observed under a microscope at 0 h and 36 h. The cell migration area was measured with ImageJ software, and the area was normalized to that of control cells.

### Transwell assay

NPC cells were resuspended in serum-free medium with or without 30 ng/ml recombinant human (rh) VEGF (1×10^5^ cells/well) and then added into the upper chambers of 24-well transwell inserts with Matrigel (invasion assay) or without Matrigel (migration assay). Medium with 10% FBS was added to the lower chambers. Twenty-four hours later, cells in the upper chambers were removed with cotton swabs, 4% paraformaldehyde was used for fixation, and Giemsa was used to stain the cells on the lower surface. Cells from random five fields in each well were imaged and counted.

### Collection of conditioned medium (CM) and tube formation assay

NPC cells with stable VEGF knockdown were cultured in complete medium until they reached 80% confluency and then washed and serum starved for 24 h. Then, CM was collected, filtered and stored at -80°C until use. Human umbilical vein endothelial cells (HUVECs) were serum starved for 6 h, resuspended in CM supplemented with 1% FBS (1×10^4^ cells/well), and added to 96-well plates that were precoated with 50 μl/well of Matrigel. The capillary network was imaged after 6 h, and the number of branch points of three random fields were counted.

### Enzyme-linked immunosorbent assay (ELISA)

NPC cells with stable VEGF knockdown were cultured without serum for 24 h, the supernatants were collected, and secreted VEGF was detected by ELISA according to the manufacturer's protocol.

### Xenograft mouse model and *in vivo* metastasis assay

Male athymic 4 to 5-week-old BALB/C nude mice were purchased from Beijing Vital River Laboratory Animal Technology Co., Ltd. and were housed in the Experimental Animal Center of Guangxi Medical University under specific pathogen-free (SPF) conditions. For the tumour growth model, stably transfected 5-8F cells (5×10^6^) were subcutaneously injected into the right lower groin of nude mice. The length and width of the xenograft tumour were observed every three days, the nude mice were sacrificed after 24 days, and the following formula was used to calculate tumour volume: V=width^2^×length×0.5. For the lung metastasis model, the indicated cells (2×10^6^) were slowly injected into nude mice through the tail vein while monitoring the vital signs of the nude mice. After 60 days, the mice were sacrificed and lung tissues were paraffin-embedded for haematoxylin and eosin (H&E) staining.

### Immunohistochemistry (IHC)

Slides with paraffin-embedded tissues were baked in a 60°C incubator for 2 h, and then after dewaxing, hydration, and antigen recovery, the slices were incubated with primary and secondary antibodies. Then, all samples were stained, dehydrated, rinsed and sealed. The immunohistochemically stained tissue sections were independently evaluated and labelled by two pathologists, and differences were resolved by reaching a consensus. The staining was categorized into different intensities: 0 (no staining), 1+ (light yellow), 2+ (yellow), and 3+ (brownish yellow). The following formula was used to calculate the H-score: [1×(% cells 1+) + 2×(% cells 2+) + 3×(% cells 3+)] × 100. When the H-score reached the maximum value of 300, 100% of the cells had strong staining intensity.

### Statistical analysis

All data are expressed as the means ± standard deviations of at least three independent experiments. Results were analysed using SPSS 17.0 or GraphPad Prism 5.0 software. Statistical *p* values were analysed by Student's *t* tests or one-way ANOVAs. A *P*-value of <0.05 was considered statistically significant.

## Results

### VEGF promotes NPC cell migration and invasion *in vitro*

**Figure 1:**
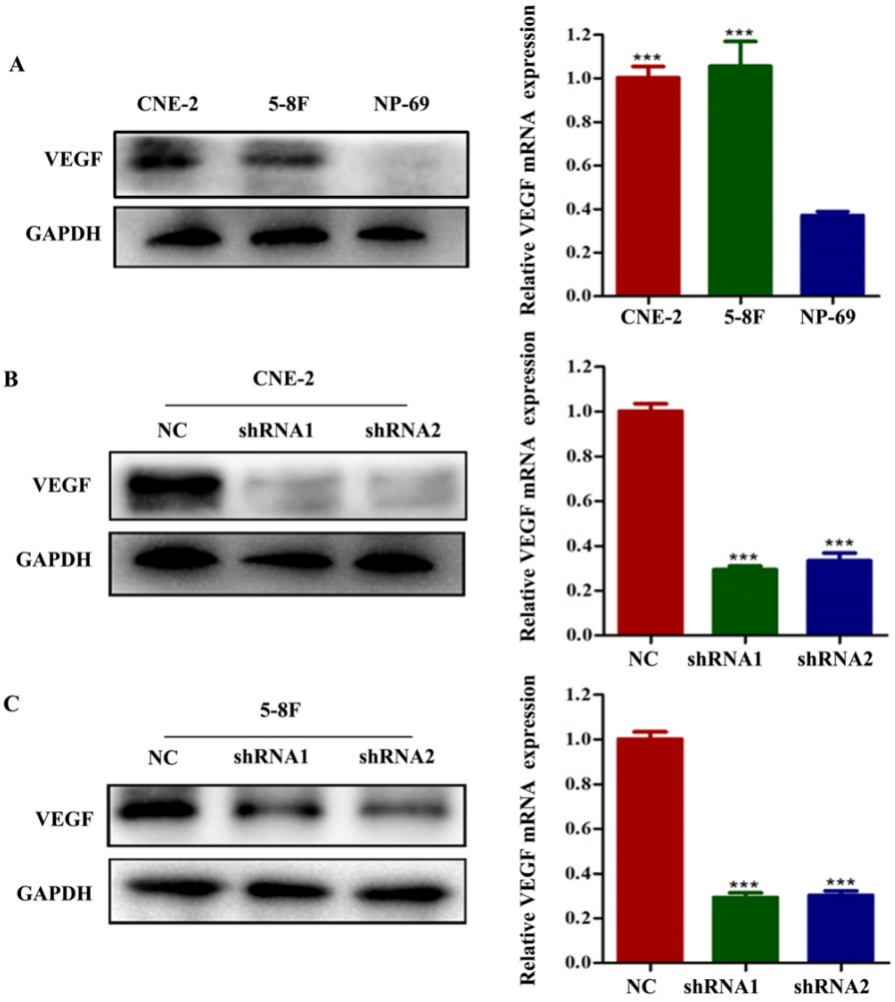
VEGF is upregulated in NPC cells, and VEGF is knocked down in NPC cells. (A) The expression of VEGF protein and mRNA in NPC cells and NP-69 cells. (B). The expression of VEGF protein and mRNA in CNE-2 cells treated with NC or VEGF shRNAs. (C) The expression of VEGF protein and mRNA in 5-8F cells treated with NC or VEGF shRNAs. *** *p*<0.001 versus NC.

To examine the potential effect of VEGF on NPC, we first analysed VEGF expression using CNE-2 and 5-8F cells and nasopharyngeal immortalized epithelial NP69 cells by western blotting and RT-qPCR. We observed that VEGF was upregulated in NPC cells (Figure 1A, all *P*<0.001); thus, we suspected that VEGF may be necessary for NPC. To test this hypothesis, NPC cells were transfected with two different short hairpin RNAs (shRNAs) targeting VEGF: 5'-GCGCAGCTACTGCCATCCAAT-3' and 5'-CACAACAAATGTGAATGCAGA-3'. The negative control (NC) sequence was 5'-TTCTCCGAACGTGTCACGT-3'. Western blot and qPCR results showed that we successfully silenced VEGF in NPC cells (Figure 1B and C, all *P*<0.001).

**Figure 2:**
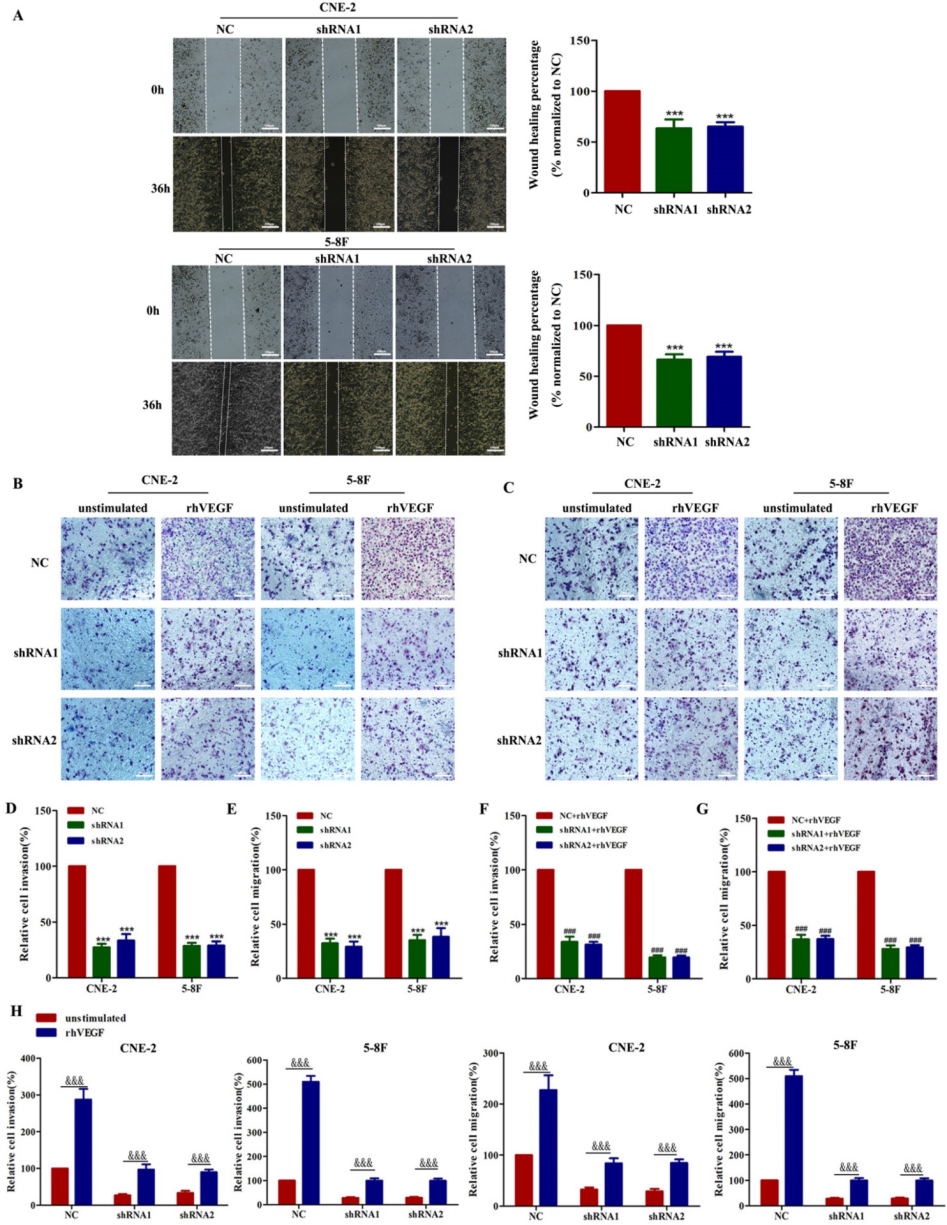
VEGF promotes NPC cell migration and invasion *in vitro*. (A). A wound healing assay was used to examine the migration ability of CNE-2 and 5-8F cells treated with NC or VEGF shRNAs (magnification, ×200), and the wound healing percentage of the NC group and shRNA group was compared 36 h and 0 h after scratching. (B) The invasion ability of VEGF-knockdown or NC cell lines with or without rhVEGF treatment was detected by a transwell assay (magnification, ×200). (C) The migration ability of VEGF-knockdown or NC cell lines with or without rhVEGF treatment was assessed by transwell assays (magnification, ×200). (D,E) Histogram is shown for invasive and migratory cells relative to NC cells. (F,G) Histogram is shown for invasive and migratory cells relative to NC cells with rhVEGF stimulated. (H) Histogram is shown for invasive and migratory cells relative to unstimulated cells. *** *p*<0.001 versus NC; ^###^ *p*<0.001 versus NC+rhVEGF; ^&&&^ *p*<0.001 versus unstimulated. Scale bar=100 µm.

As VEGF is crucial for tumour metastasis, we first performed wound healing and transwell migration assays to demonstrate that silencing VEGF inhibits cell migration in shRNA-transfected cells compared with the corresponding controls (Figure 2A, C and E, all *P*<0.001). Furthermore, transwell Matrigel invasion assays showed that silencing VEGF inhibited cell invasion in shRNA-transfected cells compared with the corresponding controls (Figure 2B and D, all *P*<0.001). Taken together, these results indicated that VEGF promoted NPC cell migration and invasion *in vitro*.

### VEGF promotes NPC cell metastasis by regulating EMT and MMPs

**Figure 3:**
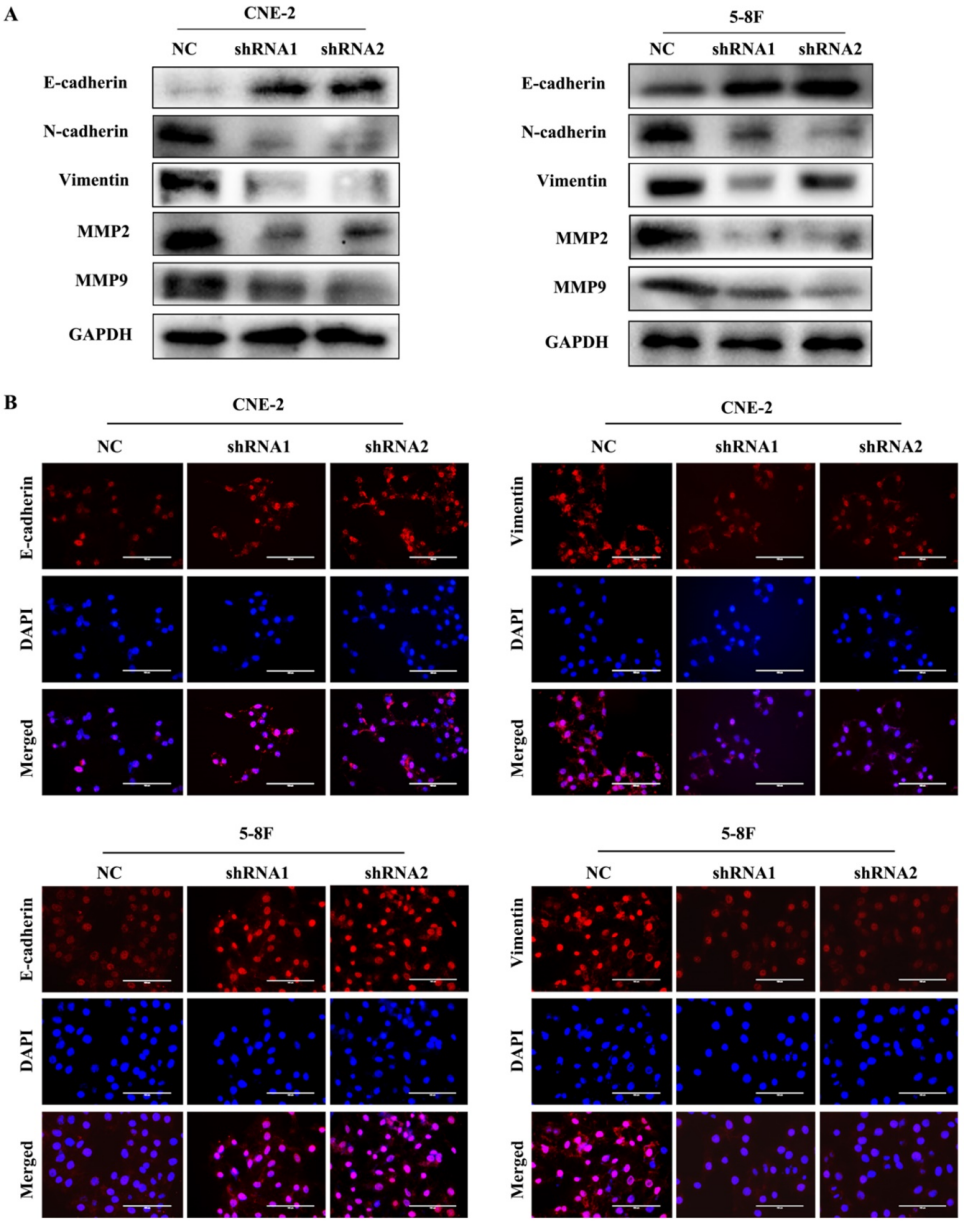
VEGF promotes NPC cell metastasis by regulating EMT and MMPs. (A) Western blotting was used to measure the metastasis-correlated proteins E-cadherin, N-cadherin, vimentin, MMP2 and MMP9 after VEGF silencing. (B) IF was used to detect E-cadherin and vimentin protein expression after VGEF silencing. Scale bar = 100 µm.

EMT is characterized by the inhibition of E-cadherin, which is a gene of epithelial connection, and the activation of vimentin and N-cadherin, which can promote interstitial adhesion 24. EMT and MMPs are critical for the metastasis of tumour cells; thus, we examined the expression levels of EMT and MMPs markers, such as E-cadherin, vimentin, N-cadherin, MMP2 and MMP9, to explore their correlation with VEGF. We found that the level of E-cadherin greatly increased in VEGF-silenced NPC cells. In contrast, the levels of N-cadherin, vimentin, MMP2 and MMP9 were markedly decreased based on western blotting (Figure 3A). Additionally, the same results were obtained in IF assays (Figure 3B). Our results revealed that VEGF promoted NPC cell metastasis by regulating the expression of EMT markers and MMPs.

### VEGF secretion enhances angiogenesis, cell migration and invasion *in vitro*

**Figure 4:**
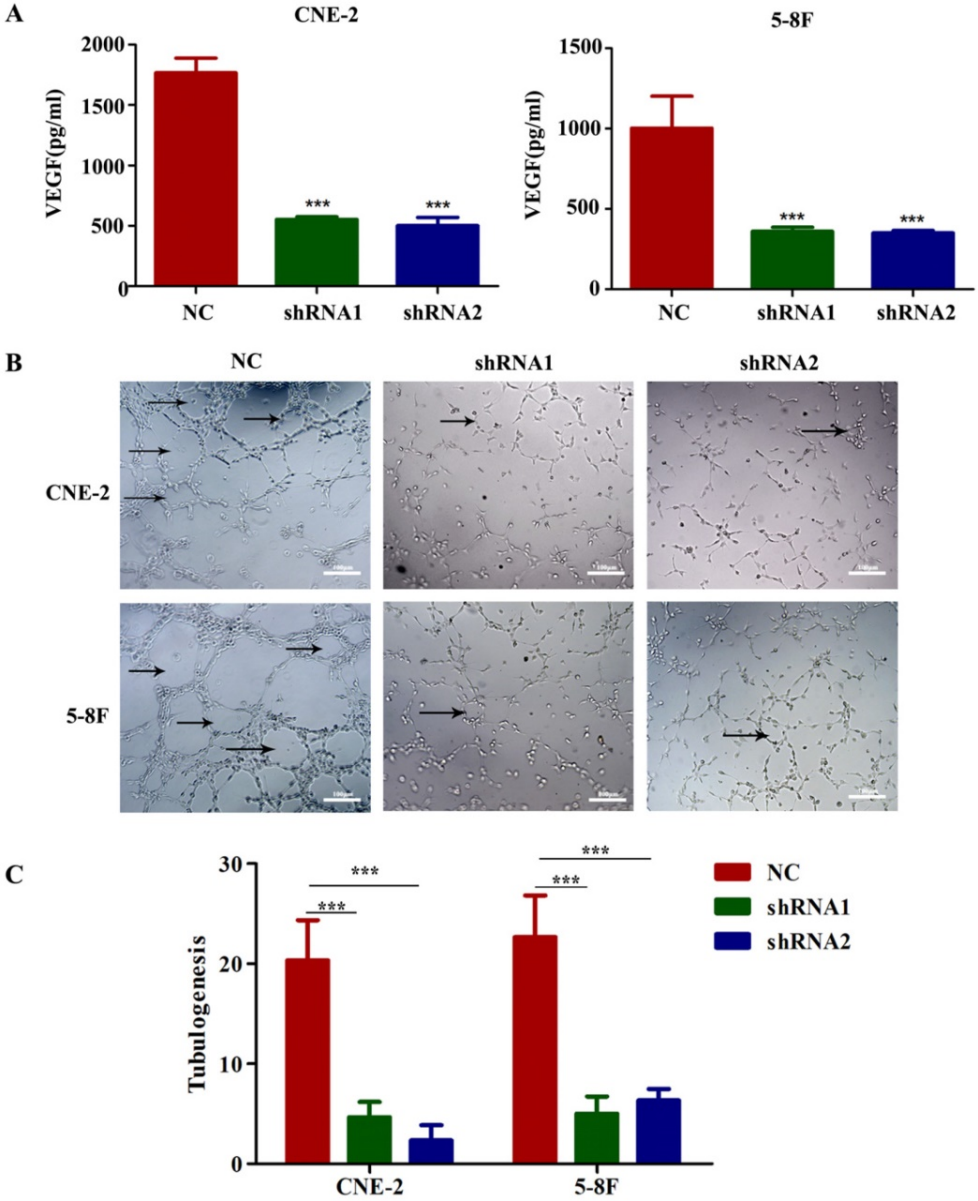
NPC cells secrete VEGF to induce angiogenesis. (A) VEGF secreted by CNE-2 and 5-8F cells treated with NC or VEGF shRNAs was measured by ELISA. (B, C) Representative images for HUVEC tube formation in response to the supernatant of CNE-2 and 5-8F cells treated with NC or VEGF shRNAs (magnification, ×200). The arrow refers to the formed blood vessel. *** *p*<0.001 versus NC. Scale bar=100 µm.

To evaluate the potential mediators of VEGF-induced metastasis in NPC cells, we first detected VEGF protein levels in NPC cell supernatants by ELISA, and the results indicated that the secretion of VEGF protein was significantly reduced in VEGF-knockdown cells (Figure 4A). Because tumour-secreted VEGF can stimulate endothelial cell angiogenesis 11, we examined tube formation by treating HUVECs with CM collected from VEGF-knockdown or NC NPC cells. As expected, CM from VEGF-knockdown cells significantly inhibited tube formation (Figure 4B and C). These findings suggest that NPC cells secreted VEGF to promote angiogenesis in a paracrine manner.

We already confirmed that the knockdown of VEGF inhibited the migration and invasion of NPC cells. Thus, we next determined whether this behaviour depended on an autocrine VEGF pathway. We found that compared with the NC group, the inhibition of VEGF continued to inhibit the invasion and migration of NPC cells in the presence of 30 ng/ml rhVEGF (Figure 2B, C, F and G). Moreover, NPC cells stimulated with rhVEGF had dramatically higher migration and invasion levels than unstimulated cells (Figure 2B, C and H), confirming that the VEGF-mediated regulation of invasion and migration required an autocrine VEGF pathway.

### VEGF regulates the expression of EMT markers and MMPs through an autocrine pathway

**Figure 5:**
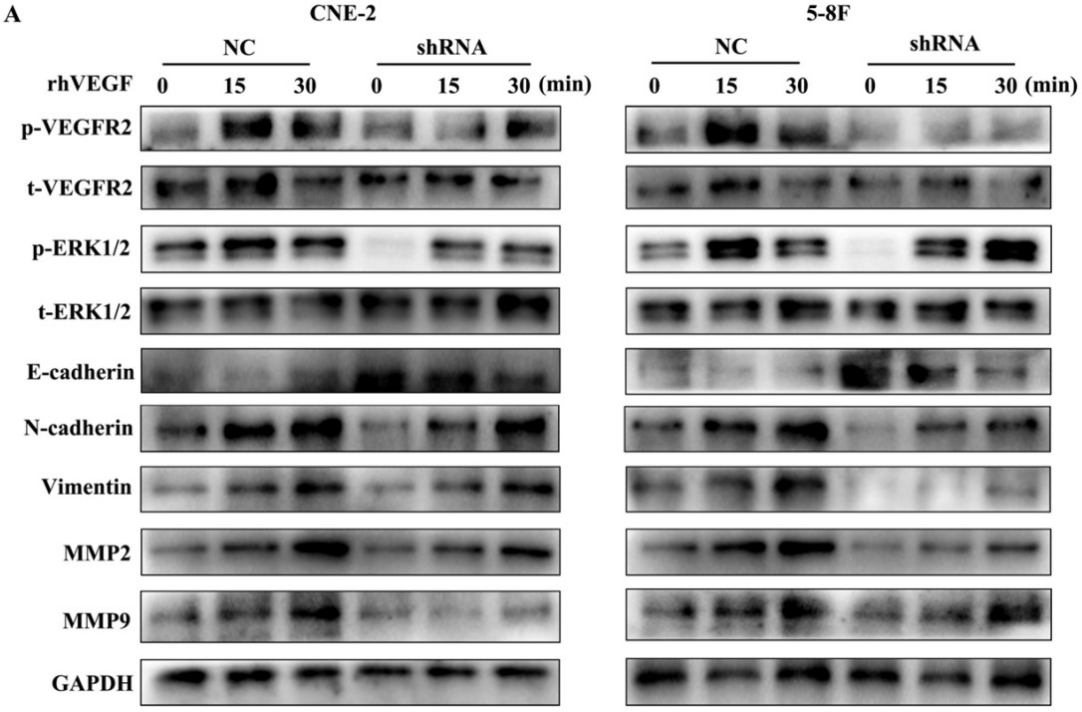
The autocrine VEGF-VEGFR2 signalling pathway promotes the expression of EMT markers and MMPs. (A) CNE-2 and 5-8F cells with stable VEGF silencing were stimulated with 30 ng/ml rhVEGF for 0, 15, and 30 min. Phosphorylation of VEGFR2 and downstream ERK1/2 and the EMT and MMP markers E-cadherin, N-cadherin, vimentin, MMP2 and MMP9 were analysed by western blotting.

As VEGFR2 is the main receptor of VEGF signalling and autocrine VEGF-VEGFR2 signalling stimulates the secretion of VEGF in diverse cell types 11, 16-18, we treated NPC cells with rhVEGF (30 ng/ml) for 0, 15, and 30 min to examine the effects of the VEGF-VEGFR2 signalling pathway on EMT and MMPs. As shown in Figure 5, phosphorylated VEGFR2 and p-ERK, a downstream kinase, were higher with rhVEGF-stimulated cells than unstimulated cells. Furthermore, treatment with rhVEGF increased the expression of N-cadherin, vimentin, MMP2 and MMP9 and decreased E-cadherin levels in NPC cells. Interestingly, even with rhVEGF treatment, the EMT and MMP protein levels of VEGF-knockdown cells were lower than those of NC cells. These data suggested that VEGF regulated the levels of EMT markers and MMPs via an autocrine pathway.

### VEGF promotes NPC tumour growth and metastasis *in vivo*

**Figure 6:**
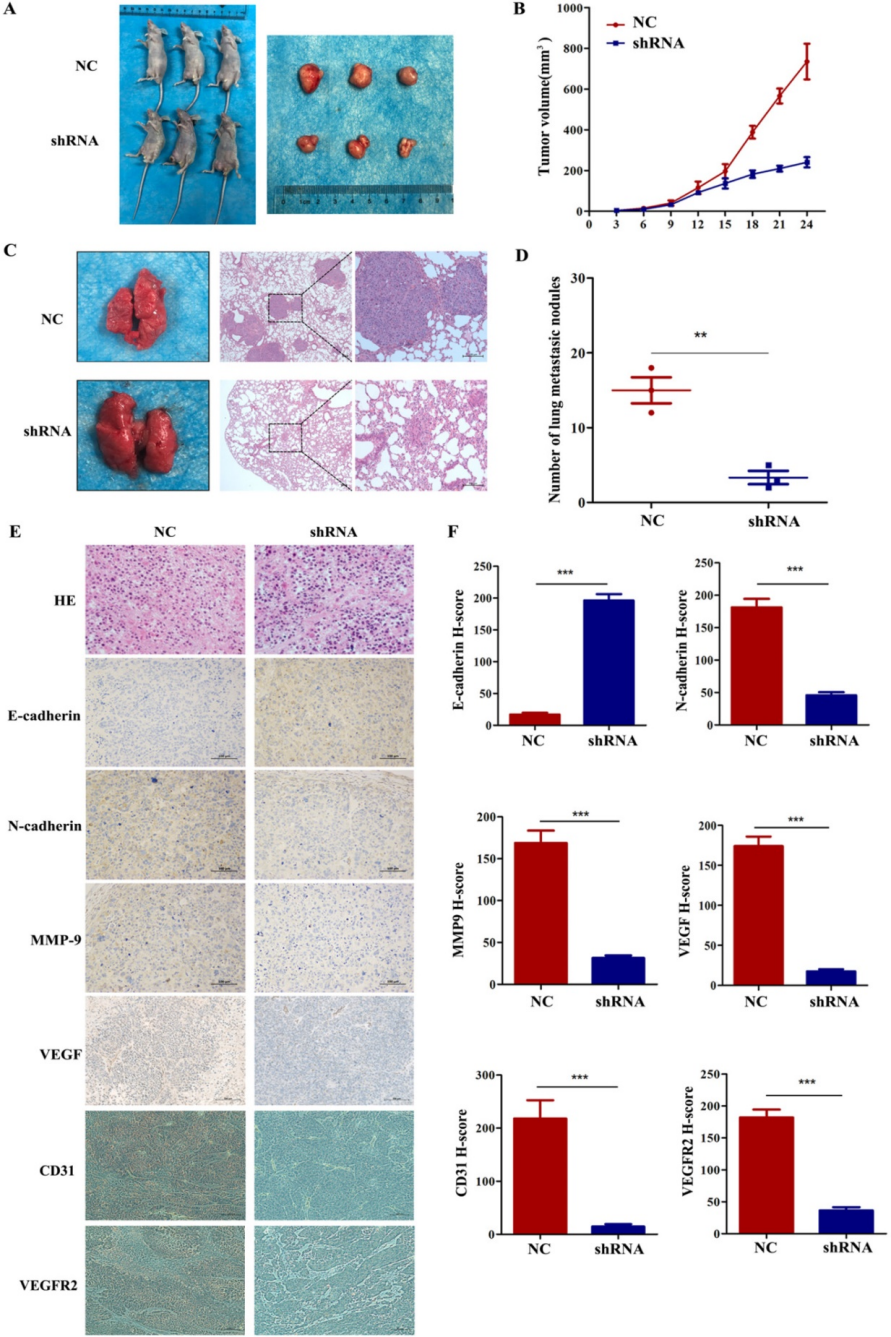
VEGF promotes NPC cell growth and metastasis *in vivo*. (A, B) Tumour size after the subcutaneous injection of NPC cells with silenced VEGF into nude mice. (C) The gross appearance of lung tissues and H&E staining for different groups of pulmonary metastatic nodules. (D) Statistical analysis of the number of pulmonary metastatic nodules in the two groups. (E) H&E staining and E-cadherin, N-cadherin, MMP9, VEGF immunostaining in two groups of tumour samples. (F) Statistical analysis of the H-score for E-cadherin, N-cadherin, MMP9, VEGF, CD31 and VEGFR2. ** *p* <0.01 versus NC, *** *p*<0.001 versus NC. Scale bar=100 µm.

To further explore whether VEGF promoted tumour progression and metastasis *in vivo*, we subcutaneously injected 5-8F cells with stably silenced VEGF into the right groin or via the tail vein of nude mice. In the tumour growth model, Figure 6A and B show that the tumours in the shRNA group were remarkably smaller than those in the NC group. In addition, IHC assays indicated that the level of E-cadherin greatly increased and the levels of N-cadherin, VEGFR2, MMP9 and VEGF remarkedly decreased in the shRNA group (Figure 6E and F, all *P*<0.001). Furthermore, we also found that the expression of CD31 decreased after VEGF was silenced (Figure 6E and F, all *P*<0.001). As a vascular marker, CD31 is not only related to vascular density but can also induce tumour metastasis 27-30. In the lung metastasis model, we found that the number of lung metastatic nodules in the shRNA group was significantly lower than that in the NC group (Figure 6C and D,* P*<0.01). Generally, these data demonstrated that VEGF promoted tumour progression and lung metastasis *in vivo*, which agrees with the *in vitro* results.

## Discussion

With the combination of magnetic resonance imaging (MRI), intensity-modulated radiation therapy (IMRT) and concurrent chemo-radiotherapy, the therapeutic efficacy of NPC patient treatment has greatly improved. It must be noted, however, that the prognosis of advanced NPC patients is poor, and the most important factor is the high rate of metastasis 31, 32. Therefore, advances in the identification of predictive biomarkers and the elucidation of underlying mechanisms are essential for more personalized treatment of NPC patients. Here, we found that VEGF was upregulated in NPC cells compared with immortalized nasopharyngeal epithelial cells. This finding suggested that VEGF may play a carcinogenic role in the development of NPC.

Most malignant tumour cells secrete VEGF, which has far-reaching effects on the progression and prognosis of tumour patients 33. VEGF plays a crucial role in promoting angiogenesis and increasing tumour cell proliferation 34, which is the main reason why VEGF can promote tumour growth and metastasis 35. Farzaneh et al 36 showed that interfering with the interaction of VEGFs and VEGFR effectively inhibited angiogenesis and tumour growth. Li et al 37 reported that VEGF expression led to colon cancer metastasis. In addition, in gastric cancer, VEGF expression is also correlated with TNM staging and lymph node metastasis 38. Moreover, distant metastasis and poor prognosis are related to VEGF in osteosarcoma, ovarian cancer and breast cancer 39-41. In this study, we found that silencing VEGF inhibited NPC cell migration and invasion, and our *in vivo* study also illustrated the correlation of VEGF with cell proliferation and lung metastasis in NPC. In addition, it would be better to study the lung metastasis models with fluorescence imaging in living animal technology, but our equipment and technical problems limited that.

During the process of metastasis, EMT can transform adherent epithelial cells into highly mobile mesenchymal cells 42. This process exacerbates the motility, infiltration and metastasis of tumour cells. The inhibition of epithelial genes and the activation of mesenchymal genes usually lead to the activation of EMT process at the transcription level 43, 44. E-cadherin is a characteristic molecular marker in epithelial cells and is located on the adhesion junction and basolateral plasma membrane 42. In addition, vimentin and N-cadherin are mainly expressed in cells with a mesenchymal origin and are closely related to tumour cell invasion 45, 46. It has been reported that increased VEGF expression in malignant cells during the transition from prostatic intraepithelial neoplasia to invasive carcinoma leads to EMT 26. Similarly, EMT proteins are often connected with MMPs in tumour cells and affect metastasis 47-50. MMPs are major proteolytic enzymes involved in tumour cell metastasis 47. The overexpression of MMPs and VEGF can lead to malignant behaviour and are correlated with poor prognosis in many tumours 51-54. Accordingly, in our study, we also found that silencing VEGF in NPC cells led to a decrease in N-cadherin, vimentin and MMPs and increase in E-cadherin, suggesting that VEGF can affect metastasis via EMT and MMPs in NPC cells.

Emerging findings have demonstrated that autocrine VEGF-VEGFR2 signalling serves as an angiogenic modulator that controls tumorigenesis and malignant behaviour in many cancer cells, including ovarian cancer and skin epithelial cancer 11, 16-18, 21. In our study, we found that NPC cells secreted VEGF and promoted angiogenesis in a paracrine manner, further supporting the role of VEGF in tumour progression. Moreover, we showed that stimulation with rhVEGF increased the migration and invasion of NPC cells, and this finding does not contradict the inhibition caused by VEGF silencing. Evidence shows that VEGF often interacts with VEGFR2, which continues to be phosphorylated and activates pERK1/2 in cells 55. Here, we found that rhVEGF induced the activation of VEGFR2 and pERK1/2 in NPC cells, and VEGF silencing attenuated these increases. In prostate cancer, VEGF has been shown to induce EMT through an autocrine pathway 26, and the VEGF autocrine loop also stimulates the secretion of MMP2 and MMP9 in childhood acute lymphoblastic leukaemia 56. In the present study, we demonstrated that NPC cells had increased expression of MMP2 and MMP9 after rhVEGF stimulation, with or without VEGF silencing. Taken together, our results showed that VEGF regulated the expression of EMT markers and MMPs and promoted NPC cell metastasis through an autocrine mechanism.

## Conclusion

We found that VEGF expression was upregulated in NPC cells. Autocrine VEGF-VEGFR2 signalling promoted EMT process and increased the expression of MMPs, leading to promoted cell invasion, migration and progression. VEGF also stimulated angiogenesis in a paracrine manner in NPC. These results demonstrate that VEGF plays an important role in tumour metastasis in NPC and holds a promising prospect for application of VEGF/VEGFR pathway inhibitors in the treatment of NPC.
